# Pain Management during Rehabilitation after Distal Radius Fracture Stabilized with Volar Locking Plate: A Prospective Cohort Study

**DOI:** 10.1155/2018/5786089

**Published:** 2018-11-05

**Authors:** Pengbo Luo, Jinjie Lou, Shengwu Yang

**Affiliations:** ^1^Department of Orthopaedic Surgery, Shanghai Jiaotong University Affiliated Sixth People's Hospital, Shanghai 200233, China; ^2^Department of Orthopedics, First Affiliated Hospital of Wenzhou Medical University, Nanbaixiang, Ouhai, Wenzhou, Zhejiang 325000, China

## Abstract

**Introduction:**

Internal fixation with volar locking plate (VLP) was widely adopted as a first-line choice in treatment of distal radius fracture (DRF).

**Methods:**

Total 315 patients with distal radius fracture receiving VLP fixation were included for analysis in this study. The rehabilitation protocol was started immediately after surgery for all patients. During the initial two weeks after surgery, 149 patients received 200 mg celecoxib twice per day, 89 received buprenorphine transdermal patch at 5 *μ*g/h, and 77 received 13 mg codeine plus 200 mg ibuprofen twice per day for pain management. Visual analog scale (VAS) scores of pain at rest, daily activity, and rehabilitative exercise were measured, respectively, every week according to the experiences of the past week in the initial six weeks after surgery. Functional outcomes including range of motion (ROM) for extension, flexion, pronation, supination, ulnar and radial abduction, the disabilities of arm, shoulder, and hand (DASH) score and the validated patient rated wrist evaluation (PRWE), and grip strength were collected at one, three, and six months after surgery.

**Results:**

We showed that patients receiving transdermal buprenorphine and codeine/ibuprofen had decreased VAS scores during rehabilitative exercise, better compliance to the rehabilitation program, and thus faster functional recovery.

**Conclusions:**

We recommend transdermal buprenorphine or codeine/ibuprofen for pain management during rehabilitation after distal radius fracture stabilized with VLP.

## 1. Introduction

Distal radius fractures are a common type of fractures, affecting more than 1% of the overall population [1]. Distal radius fractures can be treated nonoperatively if the bone fragments can be reduced to an anatomical alignment and stabilized by a plaster cast or orthotic. If this is not possible, surgical fixation is commonly recommended to be performed.

Kirschner wire (K-wire) and open reduction with VLP are the two most common surgical fixation techniques used to manage distal radius fractures. Several clinical trials have shown faster recovery of hand and wrist function with volar plating compared with K-wire or external fixation [2, 3]. As a result, the past decade shows a trend toward open reduction and internal fixation in treatment of distal radius fractures by VLP and away from K-wire and external fixation [4, 5].

K-wire fixation commonly requires postoperative immobilization to prevent secondary displacement. Hence, early mobilization and faster functional recovery are believed to be the most important advantage of VLP fixation [6, 7]. The rehabilitation program started within one week postoperatively was recommended by multiple studies [8, 9]. During postoperative rehabilitation, especially the immediate period after surgery, most of patients experience significant pain when active and passive motions are performed. To increase the tolerance to rehabilitation program, pain management is clearly important. However, there are relatively few studies in the literature that specifically evaluate pain management protocols during this stage.

## 2. Methods

The work has been reported in line with the STROCSS criteria [10].

### 2.1. Consent and Ethic Approval

The study was approved by the Ethics Committee of First Affiliated Hospital of Wenzhou Medical University. Informed consent was obtained from all participants and all methods in this study were in accordance with the Declaration of Helsinki. The study is registered in Research Registry (https://www.researchregistry.com). The registry number is researchregistry4070.

### 2.2. Participants

Total 351 patients with acute distal radius fractures (≤3 days) treated with VLP fixation from February 2014 to February 2016 were finally enrolled into this study. Exclusion criteria were age below 18 year (n=21); unwilling to participate (n=84); any concurrent injury to the upper limbs (n=23); previous wrist fracture on any side (n=7); preexisting inflammatory joint condition (n=9); professional athlete (n=3); any concurrent fracture (n=37); chronic pain or receiving pain medication (n=33); a distal radius fracture for which, in the surgeon's judgment, fixation of fracture fragments could not be achieved to allow early mobilization (n=31). Finally, 315 patients completed the six-month follow-up. The basic characteristics were summarized in Table 1. The fracture was classified according to AO Distal Radius Fracture Classification [11] (A1: extraarticular, ulnar fracture with intact radius; A2: extraarticular, radius fracture, simple or impacted; A3: extraarticular multiple fragmented fracture; B1: partial articular fracture in sagittal plane; B2: partial articular fracture in coronal plane with dorsal fragment; B3: partial articular fracture in coronal plane with volar fragment; C1: complete articular fracture, articular simple and metaphyseal simple; C2: complete articular fracture, articular simple but multiple fragmented metaphyseal; C3: complete articular fracture, multiple fragmented articular and metaphyseal).

### 2.3. Surgical Procedures and Rehabilitation Protocol

The standard volar-radial approach over the flexor carpi radialis tendon was chosen. Reduction was achieved by temporary K-wires and the fracture was then stabilized with DVR® volar plate (Zimmer Biomet). No case received bone graft in this study. Pronator quadratus repair was performed in all cases. Patients were discharged from our hospital on second to fourth postoperative day. All patients received the same standardized home exercise therapy and written physiotherapy advice. The rehabilitation protocol started immediately after surgery. The rehabilitation protocol was designed based on the previous literature [9, 12, 13] and already in place at our institution prior to this study. The detailed protocol is shown in Supplementary Table 1. Whether each patient completed the rehabilitation program was monitored by therapist through phone calls on weekly basis.

### 2.4. Pain Management and Assessment

Due to the lack of evidences, the pain management protocols were designed based on our previous experiences and currently available literature on chronic pain management [14, 15]. There were three in-use protocols which were 200 mg celecoxib twice per day; buprenorphine transdermal patch at 5 *μ*g/h; 13 mg codeine plus 200 mg ibuprofen twice per day. The duration of pain medication was two weeks after surgery for all three protocols. Patients were grouped based on the received pain medication (celecoxib, transdermal buprenorphine, and codeine/ibuprofen).

Visual analog scale (VAS) score ranges from 0 (no pain) to 100 mm (worst pain possible). VAS score was measured every week according to the patient's experience of average pain in the past week in the initial six weeks after surgery. The VAS scores in three different scenarios (resting, daily activity, and rehabilitative exercise) were measured.

### 2.5. Outcome Measurement

Range of motion (ROM) for extension, flexion, pronation, supination, and ulnar and radial abduction was documented for both the healthy and the injured wrist using a goniometer. The ROM in this study was presented as a percentage calculated according to the ROM of uninjured side. Because there are interindividual variation in mobility, the ROM of the uninjured side was defined as the standard value and set as 100%. The disabilities of arm, shoulder, and hand (DASH) score are a 30-item self-report questionnaire (0-100, with 100 indicating greater disability) which provides a general measure of physical function and symptoms in people with disorders of the upper limb. The validated patient rated wrist evaluation (PRWE) rates wrist function in two equally weighted sections concerning the patient's experience of pain and disability. The outcome is a score out of 100 (with 100 indicating the worst condition). Grip strength was measured with the elbow flexed at 90 degree and the forearm in neutral rotation. Values are expressed as percentages of the values of the contralateral hand. The PRWE, DASH, grip strength, and ROM of wrist were collected at one, three, and six months after surgery.

## 3. Results

Total 351 patients with acute distal radius fractures treated with VLP fixation from February 2014 to February 2016 were enrolled into this study. Finally, 315 patients completed the six-month follow-up. Among patients who completed the entire follow-up, 149 patients received celecoxib, 89 patients received transdermal buprenorphine, and 77 patients received codeine/ibuprofen during the initial two weeks postoperatively. Loss to follow-up occurred in 17, 10, and 9 patients of celecoxib, transdermal buprenorphine, and codeine/ibuprofen groups, respectively. The basic characteristics were summarized in Table 1.

### 3.1. Outcomes of Pain Management during Rehabilitation Program

Shown in Table 2, the pain at rest and daily activity among all three groups was mild (< 30 mm) and similar in all six postoperative time points. However, the difference in VAS scores at rehabilitative exercise among the groups was statistically significant (*P* < 0.05) within two weeks postoperatively. Celecoxib at 200 mg per day showed inferior effects in pain management during rehabilitative exercise compared with the other two groups. Consistently, transdermal buprenorphine and codeine/ibuprofen increased the patients' compliance to the rehabilitation program. The percentage of patients in celecoxib group who completed the rehabilitative program was the lowest within the initial two weeks. The difference in VAS scores at rehabilitative exercise in Week 6 was statistically significant (*P* = 0.04) but not clinically relevant.

### 3.2. Functional Outcomes

**Figure 1:**
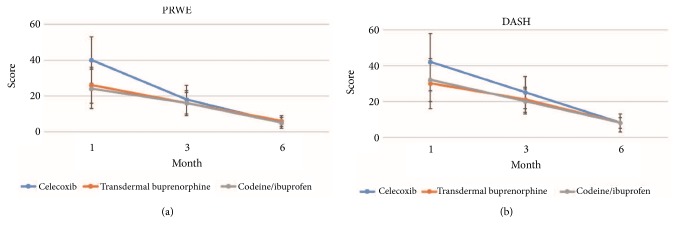
PRWE and DASH scores at one, three, and six months postoperatively.

As expected, the recovery of celecoxib group was significantly slower than that of the other groups (Figure 1 and Table 3). PRWE and DASH scores of celecoxib group were significantly lower at one month and three months. ROM for extension, flexion, supination, and ulnar and radial abduction of celecoxib group was inferior at one and three months as well. Notably, the differences in radial and ulnar abduction were still significant at the end of follow-up while the differences in the other parameters were no longer significant. Grip strength and pronation among the three groups were similar at all time points.

## 4. Discussion

The concept that early rehabilitation after fractures could accelerate functional recovery has been well-established among orthopedic surgeons and therapists. The rehabilitation program of distal radius fractures stabilized with VLP was recommended to be started within one week by multiple studies [8, 9]. However, most of patients experience significant pain when active and passive exercises are conducted during rehabilitation, especially in the early stage, which reduces patients' compliance to the rehabilitation program. However, there are relatively few evidences that specifically evaluate pain management protocols during this stage. The current practices of pain management in this phase were empirical.

There were three in-place protocols using celecoxib, transdermal buprenorphine, and codeine/ibuprofen respectively. All three protocols showed satisfactory pain relief at rest and daily activity with no significant difference. However, patients receiving buprenorphine transdermal patch and codeine plus ibuprofen experienced less pain during rehabilitative exercise compared with those receiving celecoxib. As a result, transdermal buprenorphine and codeine/ibuprofen groups had a significantly faster recovery compared with celecoxib group.

The duration of pain medication was two weeks after surgery for all three protocols. The VAS scores of Week 3 at rest, daily activity, and exercise were all quite mild, indicating it was not necessary to further prolong the duration of pain medication. It is still not clear whether the duration could be shortened.

Several limitations should be considered when interpreting the study results. As all observational studies where the treatment is not randomly assigned, unmeasured confounding factor could possibly exist and bias the study results. In clinical setting, patients with more pain are commonly given a stronger pain medication regimen. The different physicians also have different preferences on postoperative pain management. In addition, participants of this study were aware of their treatment and their expectations of pain relief could be different. In addition, we did not collect the information of opioid and NSAID utilization and thus was unable to provide more useful information. Finally, this study only addressed the comparative effectiveness of the three in-use protocols in our hospital. We did not make a comparison to no analgesics.

## 5. Conclusions

We recommend transdermal buprenorphine or codeine/ibuprofen for pain management during rehabilitation after distal radius fracture stabilized with VLP.

## Figures and Tables

**Table 1:** Basic characteristics of 315 study participants by treatment group.

	Celecoxib (n=149)	Transdermal buprenorphine (n=89)	Codeine/ ibuprofen (n=77)	*P* value
Mean (SD) age (years)	55.7 (15.4)	54.6 (16.1)	54.9 (17.2)	0.82
Sex				
Male	34 (23)	21 (24)	19 (25)	0.95
Female	115 (77)	68 (76)	58 (75)	
Side of injury				
Left	78 (52)	49 (55)	39 (51)	0.85
Right	71 (48)	40 (45)	38 (49)	
Handedness of patient				
Left	22 (15)	12 (13)	13 (17)	0.83
Right	127 (85)	77 (87)	64 (83)	
Fracture classification*∗*				
A1, A2, A3	0, 47, 50 (65)	0, 28, 28 (63)	0, 25, 26 (66)	0.99
B1, B2, B3	2, 3, 2 (5)	2, 1, 1 (4)	1, 1, 1 (4)	
C1, C2, C3	18, 20, 7 (30)	13, 11, 5 (33)	10, 9, 4 (30)	
Long-term alcohol consumption	23 (15)	13 (15)	16 (21)	0.50
Smoker	25 (17)	19 (21)	13 (17)	0.64

Figures are numbers (percentage) unless stated otherwise*∗*.

**Table 2:** VAS scores (mm) measured every week during rehabilitation program.

	Celecoxib (n=149)	Transdermal buprenorphine (n=89)	Codeine/ ibuprofen (n=77)	*P* value
Week 1				
VAS score (rest)	27 (8)	26 (8)	28 (8)	0.35
VAS score (daily activity)	31 (10)	32 (10)	33 (11)	0.66
VAS score (rehabilitative exercise)	67 (22)^#^	45 (14)^*∗*^	42 (12)^*∗*^	< 0.01
Completing the rehabilitation program	80 (54)	68 (76)	57 (74)	< 0.01
Week 2				
VAS score (rest)	24 (7)	22 (7)	23 (6)	0.29
VAS score (daily activity)	26 (9)	25 (8)	24 (10)	0.34
VAS score (rehabilitative exercise)	41 (17)^#^	30 (11)^*∗*^	32 (10)^*∗*^	< 0.01
Completing the rehabilitation program	110 (74)	76 (85)	66 (86)	0.03
Week 3				
VAS score (rest)	19 (6)	18 (5)	18 (6)	0.11
VAS score (daily activity)	22 (7)	20 (7)	21 (7)	0.10
VAS score (rehabilitative exercise)	26 (8)	24 (8)	24 (8)	0.16
Completing the rehabilitation program	135 (91)	80 (90)	68 (88)	0.86
Week 4				
VAS score (rest)	15 (5)	16 (5)	16 (5)	0.37
VAS score (daily activity)	16 (6)	15 (5)	17 (8)	0.39
VAS score (rehabilitative exercise)	17 (8)	19 (9)	18 (6)	0.20
Completing the rehabilitation program	141 (95)	82 (92)	72 (94)	0.75
Week 5				
VAS score (rest)	12 (4)	12 (4)	12 (4)	0.82
VAS score (daily activity)	12 (5)	13 (6)	11 (5)	0.39
VAS score (rehabilitative exercise)	14 (5)	14 (5)	14 (7)	0.85
Completing the rehabilitation program	144 (97)	86 (97)	73 (95)	0.77
Week 6				
VAS score (rest)	6 (4)	7 (4)	7 (4)	0.11
VAS score (daily activity)	8 (4)	8 (4)	8 (4)	0.35
VAS score (rehabilitative exercise)	8 (3)	7 (3)	8 (3)	0.04
Completing the rehabilitation program	142 (95)	84 (94)	74 (96)	0.87

Pain medication was given only in Week 1 and 2. Figures are Mean (SD) for VAS score and otherwise number (percentage).

*∗P* < 0.05 versus celecoxib group; # *P* < 0.05 versus transdermal buprenorphine group. *P* value was calculated using Student's t test.

**Table 3:** Functional outcomes at one, three, and six months postoperatively.

	Celecoxib (n=149)	Transdermal buprenorphine (n=89)	Codeine/ ibuprofen (n=77)	*P* value
One month				
PRWE	40 (13)^#^	26 (10)^*∗*^	24 (11)^*∗*^	< 0.01
PRWE (pain subscale)	14 (6)	13 (5)	12 (6)	0.63
DASH	42 (16)^#^	30 (14)^*∗*^	32 (12)^*∗*^	< 0.01
Grip strength (%)	45 (13)	46 (21)	47 (20)	0.78
Extension (%)	43 (16) ^#^	61 (18) ^*∗*^	60 (15) ^*∗*^	< 0.01
Flexion (%)	60 (21) ^#^	71 (22) ^*∗*^	71 (18) ^*∗*^	< 0.01
Pronation (%)	80 (21)	79 (24)	83 (20)	0.55
Supination (%)	54 (16) ^#^	60 (22) ^*∗*^	60 (28) ^*∗*^	0.03
Ulnar abduction (%)	45 (18) ^#^	60 (15) ^*∗*^	61 (19) ^*∗*^	< 0.01
Radial abduction (%)	50 (23) ^#^	57 (21) ^*∗*^	59 (26) ^*∗*^	0.01
Three months				
PRWE	18 (8) ^#^	16 (6) ^*∗*^	16 (7) ^*∗*^	0.04
PRWE (pain subscale)	5 (2)	6 (3)	5 (3)	0.67
DASH	25 (9) ^#^	21 (7) ^*∗*^	20 (7) ^*∗*^	< 0.01
Grip strength (%)	91 (9)	91 (7)	90 (9)	0.79
Extension (%)	73 (17) ^#^	78 (20) ^*∗*^	80 (26) ^*∗*^	0.02
Flexion (%)	78 (30)	84 (22)	87 (23)	0.04
Pronation (%)	90 (10)	93 (16) ^*∗*^	91 (12) ^*∗*^	0.21
Supination (%)	60 (21) ^#^	68 (27) ^*∗*^	67 (28) ^*∗*^	0.02
Ulnar abduction (%)	65 (18) ^#^	78 (21) ^*∗*^	76 (25) ^*∗*^	< 0.01
Radial abduction (%)	68 (19) ^#^	75 (23) ^*∗*^	75 (26) ^*∗*^	0.03
Six Months				
PRWE	5( 3)	6 (3)	5 (3)	0.28
PRWE (pain subscale)	3 (2)	3 (2)	2 (1)	0.81
DASH	8 (3)	8 (5)	8 (3)	0.95
Grip strength (%)	90 (10)	89 (9)	91 (6)	0.46
Extension (%)	92 (12)	92 (10)	91 (8)	0.81
Flexion (%)	93 (11)	96 (10)	93 (9)	0.12
Pronation (%)	93 (6)	93 (8)	92 (10)	0.76
Supination (%)	90 (11)	90 (13)	88 (15)	0.26
Ulnar abduction (%)	86 (15) ^#^	91 (12) ^*∗*^	90 (13) ^*∗*^	0.01
Radial abduction (%)	88 (17) ^#^	93 (15) ^*∗*^	93 (15) ^*∗*^	0.04

Figures are mean (SD).

*∗P* < 0.05 versus celecoxib group; # *P* < 0.05 versus transdermal buprenorphine group. *P* value was calculated using Student's t-test.

## Data Availability

The data used to support the findings of this study are included within the article.

## Supplementary Materials

Supplementary Materials Supplementary Table 1: detailed description of home exercise program on a weekly schedule.
