# Supplementary material for: Pain Management during Rehabilitation after Distal Radius Fracture Stabilized with Volar Locking Plate: A Prospective Cohort Study
Source: Biomed Res Int. 2018 Nov 5;2018:5786089. doi: 10.1155/2018/5786089 (PMC6241235; doi:10.1155/2018/5786089)
Supplement: Supplementary Materials — Supplementary Table 1: detailed description of home exercise program on a weekly schedule. [file 5786089.f1.docx]

**Supplementary Table 1 Detailed description of home exercise program on a weekly schedule**

| Time point | Description | Daily frequency* |
| --- | --- | --- |
| Week 1 | Finger fully passive motion | 3 × 20 rep |
|  | Finger, Wrist, forearm, elbow, shoulder active motion | 3 × 15 rep |
| Week 2 | Wrist, forearm fully passive motion | 3 × 20 rep |
|  | Squeezing a soft foam ball | 3 × 15 rep |
| Week 3 | Light putty strengthening (0.5 kg) | 2 × 10 rep |
|  | Squeezing a soft foam ball | 3 × 15 rep |
| Week 4 | Medium putty strengthening (1 kg) | 2 × 10 rep |
|  | Squeezing a tennis ball | 3 × 15 rep |
| Week 6 | Heavy putty strengthening (2 kg) | 2 × 10 rep |
|  | Squeezing a tennis ball | 3 × 15 rep |

* Exercises were performed twice daily with a 10-second break between exercises.
